# Predictors of Facebook User Engagement With Health-Related Content for Gay, Bisexual, and Other Men Who Have Sex With Men: Content Analysis

**DOI:** 10.2196/publichealth.8145

**Published:** 2018-04-06

**Authors:** Kiffer George Card, Nathan Lachowsky, Blake W Hawkins, Jody Jollimore, Fahmy Baharuddin, Robert S Hogg

**Affiliations:** ^1^ Faculty of Health Science Simon Fraser University Burnaby, BC Canada; ^2^ British Columbia Centre for Excellence in HIV/AIDS Vancouver, BC Canada; ^3^ School of Public Health and Social Policy University of Victoria Victoria, BC Canada; ^4^ Interdisciplinary Studies Graduate Program University of British Columbia Vancouver, BC Canada; ^5^ Community-Based Research Centre for Gay Men's Health Vancouver, BC Canada; ^6^ YouthCO HIV & Hep C Society Vancouver, BC Canada

**Keywords:** social media, health promotion, gay and bisexual men, user engagement

## Abstract

**Background:**

Social media is used by community-based organizations (CBOs) to promote the well-being of gay and bisexual men (GBM). However, few studies have quantified which factors facilitate the diffusion of health content tailored for sexual minorities.

**Objective:**

The aim of this study was to identify post characteristics that can be leveraged to optimize the health promotion efforts of CBOs on Facebook.

**Methods:**

The Facebook application programming interface was used to collect 5 years’ of posts shared across 10 Facebook pages administered by Vancouver-based CBOs promoting GBM health. Network analysis assessed basic indicators of network structure. Content analyses were conducted using informatics-based approaches. Hierarchical negative binomial regression of post engagement data was used to identify meaningful covariates of engagement.

**Results:**

In total, 14,071 posts were shared and 21,537 users engaged with these posts. Most users (n=13,315) engaged only once. There was moderate correlation between the number of posts and the number of CBOs users engaged with (*r*=.53, *P*<.001). Higher user engagement was positively associated with positive sentiment, sharing multimedia, and posting about pre-exposure prophylaxis, stigma, and mental health. Engagement was negatively associated with asking questions, posting about dating, and sharing posts during or after work (versus before).

**Conclusions:**

Results highlight the existence of a core group of Facebook users who facilitate diffusion. Factors associated with greater user engagement present CBOs with a number of strategies for improving the diffusion of health content.

## Introduction

Gay, bisexual, and other men who have sex with men (GBM) are at elevated risk for a number of adverse health outcomes [1,2]. Stall et al [3] argues that gay communities experience a syndemic of co-occurring sexual, substance use, and psychosocial conditions that, according to Singer [4], work synergistically under “deleterious social and physical conditions” (p 15) to adversely affect the health of this population [5]. In response, public health and community leaders have advanced holistic approaches to gay men’s health that address not only individual and biological factors, but also the broader psychosocial and structural factors that affect their health and well-being [6].

In implementing such programs, social media is widely used by community-based organizations (CBOs) to disseminate health information and engage with GBM [7-9]. Indeed, social media has come to play a significant and diverse role in a variety of health contexts. Articulating this role, Kietzmann et al [10] highlight seven personal and interpersonal needs that social media has come to fulfill. Broadly, we summarize these needs by three activities: identity management, communication, and social bonding. In the context of GBM health, sexual minorities have always needed spaces where they can engage in these activities, and social media has come to provide such spaces [11,12].

Although the Internet provides a platform whereby CBOs can reach GBM, the success of these interventions is far from guaranteed [13]. Rogers’ Diffusion of Innovations Theory describes the challenges to CBOs in terms of diffusion, reach, and uptake [14,15]. In brief, Rogers posits that key characteristics of individuals (whom he describes as “adopters”) and the network ties that connect them to others in a social network are fundamental to the spread of information, behavior, and products. A number of factors have been identified that impact adoption and diffusion (eg, age, social network structure, personality types), and media richness theory describes how specific media (ie, routes of content delivery) detract or promote diffusion [16]. Furthermore, he argues that more “life-like” interactions better promote uptake of new ideas.

In the age of social media, specific engagement indicators (ie, reactions, comments, and shares) on Facebook provide rudimentary markers for diffusion—and, in fact, are used by Facebook’s EdgeRank algorithm to govern which messages are shown to other users [17]. Barriers to diffusion are particularly relevant to efforts targeting GBM, who represent a diverse and uniquely organized group of individuals [18]. For example, Cassidy [19] notes that campaigns to amass likes, comments, and shares can often be at odds with an individual’s need to manage their public identity. After all, not all sexual minorities openly acknowledge their sexuality online—especially in spaces where multiple social circles collide [20]. Yet, if social media strategies among GBM are to be successful, CBOs must find ways to encourage users to engage with their content. This is because many social media platforms rely on engagement-based algorithms to determine if social media content is viewed by other users. For example, according to Facebook:

The stories that show in your News Feed are influenced by your connections and activity on Facebook. This helps you to see more stories that interest you from friends you interact with the most. The number of comments and likes a post receives and what kind of story it is (ex: photo, video, status update) can also make it more likely to appear in your News Feed.

17

Consistent with this, increasing user engagement (defined by Facebook as the composite of reactions, comments, and shares on a post) has become a primary objective of social media campaigns, and a handful of studies have sought to identify predictors of user engagement. For example, Veale et al [21] identified 10 Twitter and Facebook profiles with high user engagement and found that these organizations gained prominence by posting regularly, engaging with individual users, encouraging interaction and conversation by posing questions, sharing multimedia, and highlighting celebrity involvement. In a similar study, Kite et al [22] found that higher post engagement among 20 Facebook health profiles was associated with positive sentiment, providing factual information, inclusion of videos, and celebrity marketing. Likewise, Rus and Cameron [23] explored 10 diabetes-related health pages and found that imagery was a strong predictor of engagement. Further, they identified other characteristics, such as sentiment, crowdsourcing, and providing factual information, that were associated with some, but not all, forms of engagement. However, as campaigns addressing sensitive subjects and those targeting sexual minorities might be uniquely constrained by users’ willingness to publicly endorse or share CBO-generated content, context-specific evaluations of user engagement are needed. As such, the primary objective of this study was to identify strategies to enhance user engagement.

Additionally, it is unclear whether Facebook is even an effective platform for CBOs to reach sexual minority populations [24]. Indeed, although social media campaigns might gain the attention of local network members, they may miss those who are not directly associated with CBOs. Despite widely held assumptions of Facebook’s communication potential [9], little research has been conducted on the Facebook network structure of sexual minorities. Optimistically, that which has suggests that the Facebook network structure of sexual minorities is scale-free [25], meaning that some individuals are more embedded in the social network than others and that these individuals act like “hubs” diffusing information into their local networks. However, although scale-free networks are said to effectively transmit information [26], their efficiency relies on the ways these networks are organized [14]. For example, scale-free networks with high modularity (ie, the appearance of distinct clusters or communities within a network) promote strong bonds between network members and thus saturation of local networks, whereas those with low modularity promote weak ties between individuals, but broad global diffusion [27,28]. Both modular and nonmodular network structures offer benefits and limitations; for example, experimental research by Bakshy et al [27] shows that strong ties increase the likelihood that individuals will share content shared by other network members, whereas weak ties facilitate the diffusion of information between network clusters. Therefore, as a secondary objective, this study aimed to complement our understanding of the diffusion of information through the Facebook networks of CBOs in Vancouver, British Columbia (BC).

## Methods

Consistent with these objectives, this study leveraged data collected from 10 Facebook pages (ie, all pages identified as being administered by selected organizations) belonging to eight CBOs in Vancouver, BC. Pages were purposively selected (ie, all identified organizations were included) that were (1) well known to our study team (ie, community-based partners or those otherwise highly visible), (2) inclusive of or targeted toward sexual minorities (ie, page content relevant, at least in part, to sexuality, sexual health, or community social issues), and (3) dealt primarily with health promotion (ie, health promotion was main goal of the organization).

To ensure user privacy and compliance with Facebook’s end-user agreement, data were downloaded using Facebook’s public application programming interface (API) accessed through the Netvizz Facebook app [29]. Data collected between January 1, 2010 and August 31, 2016, via Netvizz were hierarchically organized by page and post. The first year—2010—was selected based on the completion of the iPrEx trial examining the efficacy of pre-exposure prophylaxis (PrEP), one of the key topics assessed in this analysis [30]. At the page level, we identified the number of followers for each page. On the post level, we identified the number of likes, comments, and shares on each post. Netvizz also assigned unique identifiers to each user, allowing us to examine user engagement across multiple posts and multiple pages. As such, we used Spearman rank correlation to determine whether there was an association between frequency of participation and participation across multiple pages. Further, a network diagram showing the ways individuals interacted with posts from the 10 CBOs was constructed in Gephi 0.9.1 using the ForceAtlas2 layout algorithm [31]. Modularity clusters were also identified using Gephi’s modularity tool with the resolution set to 1 in order to maximize the modularity [32]. Because this study leveraged publicly available data, the research ethics board at Simon Fraser University deemed the study exempt from review. As an extra precaution on behalf of the users whose data were included in the present analysis, the names of the Facebook pages included in our study have been omitted. However, Table 1 provides a short characterization of the mission of each page to highlight the range of groups included in our analysis.

The content of each post was then analyzed using informatics-based methodology [33-35]. First, using researcher-generated search taxonomies, we identified posts relating to eight topics (with keywords for each topic in parentheses): pre-exposure prophylaxis (ie, PrEP, preexposure, pre-exposure, prophylaxis), treatment (ie, treatment, undetect*, viral load, viral-load), condoms (ie, condom*), mental health (ie, mental, emotion*, depress*, anxiety), stigma (ie, stigma, discriminat*), testing (ie, test*, screening, checked online), dating (ie, dating, relationship), and research (ie, research*, study). Posts that utilized questions to engage users were also recorded by identifying posts with a question mark (ie, “?”). Similarly, posts which directly encouraged user engagement were identified by searching for key terms inviting participation (ie, like, comment, share, take, visit).

Further, each sentence of each post was scored using the Bing Liu sentiment lexicon [34]. The Bing Liu sentiment lexicon, which is widely used in sentiment analysis and opinion mining, was selected because it provides a freely accessible word database that assigns positive and negative values to keywords, including commonly misspelled words. After each word within each sentence was scored, an average sentiment score was assigned to each post indicating whether the post had an overall negative or positive affect.

We then used multivariable hierarchical negative binomial regression to identify the post characteristics associated with greater user engagement. In this analysis, Facebook’s engagement score was used because this is presumably an important variable used in their News Feed algorithm. According to Facebook’s API, the number is calculated as the combined total number of reactions, shares, and comments on each post. Hierarchical negative binomial regression modeling was selected as the statistical approach for this study because the Facebook engagement count data were overdispersed, highly skewed toward 0 and 1, and came from 10 separate Facebook pages—each with a varying number of Facebook “fans” and with differing rates of activity. Incidence rate ratios (IRR) presented in text were calculated by exponentiating the regression coefficients. All coding and statistical analysis were conducted in RStudio.

## Results

Table 1 provides a basic description for each of the 10 Facebook pages included in our study, including the number of posts shared by each organization. Table 2 provides an overview of the posts analyzed in this study. During the study period between January 1, 2010 and August 31, 2016, 14,071 posts were shared. In total, 21,537 unique users were identified as having engaged with at least one post. Most users engaged only once (n=13,315), two to five times (n=4872), or six to nine times (n=1197). Approximately 10% (2153/21,537) of users engaged more than 10 times.

**Figure 1:**
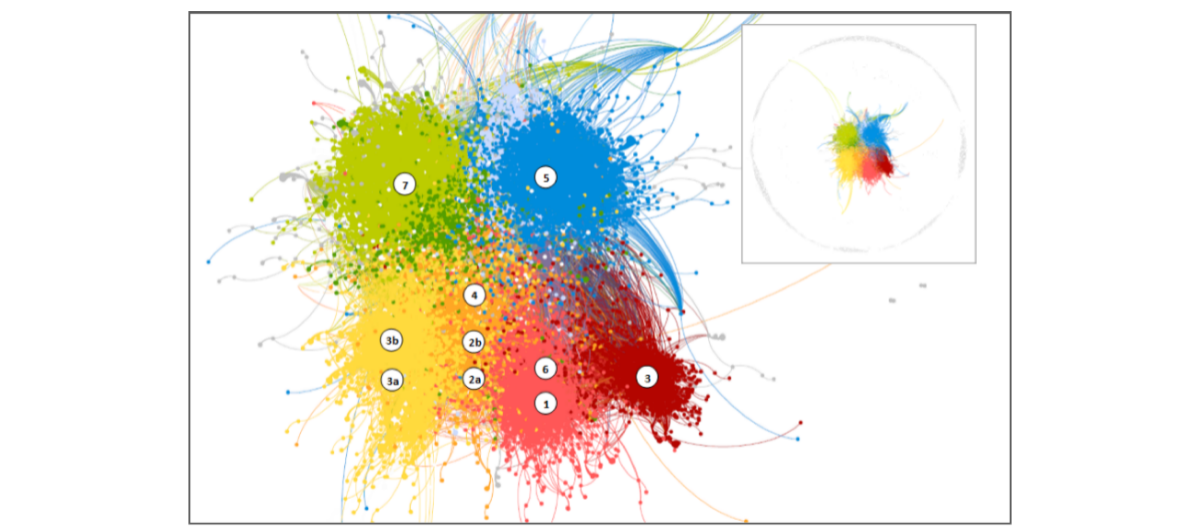
Network diagram illustrating user engagement with each post. Colors represent modularity clusters. Numbered symbols represent each Facebook page with the location indicating the modularity class in which most posts were located.

Similarly, most users engaged with content from only one (n=18,837) or two (n=1978) groups. Only a small minority of users (n=722) interacted with more than three groups. Despite low overall engagement (low number of users who “engaged” with content more than once), high modularity (Q=0.62) was observed in the ways individuals interacted with shared content (see Figure 1). Indeed, eight modularity clusters accounted for 74.49% of posts (10,481/14,071) and 93.31% of users (20,097/21,537). There was moderate correlation between the number of posts and the number of CBOs users engaged with (*r*=.53, *P*<.001).

**Table 1:** Description of selected Facebook pages serving Vancouver’s gay communities. LGBT2SQ: lesbian, gay, bisexual, transgender, two-spirited, queer. IQR: interquartile range.

ID and description of organization		Months of observation^a^	“Facebook fans”	Posts shared by organization	Engagements
1	AIDS service organization	78	1168	1458	1281
2a	Gay men’s research organization	47	422	578	262
2b	Anti-stigma social media campaign^b^	10	1126	242	524
3	AIDS fundraising organization	79	1578	699	1385
4	Gay men’s health organization	80	2290	2166	3184
5	LGBT2SQ Pride organization	74	8813	1791	7405
6	AIDS service organization	65	1015	1921	675
7	Queer community organization	80	5571	3607	9351
8a	Youth-led health organization	74	1097	758	824
8b	Peer-led program for young LGBT2SQ^b^	54	716	851	598
Median (IQR)		74 (57-79)	1147 (1036-2112)	1155 (714-1889)	1053 (617-2734)
Total		641	23,796	14,071	25,489

^a^Months of observation indicate the total number of months the page was operational for, with organizations new to the Facebook platform providing fewer months of observation.

^b^These pages are associated with the Facebook page listed before (ie, are administered by these groups as subprograms, but for marketing reasons are separate from the main page administered by the organization).

**Table 2:** Post characteristics across 10 Facebook pages serving Vancouver’s gay communities, 2010-2016. IQR: interquartile range.

Post characteristics			Facebook posts
**Post type, n (%)**			
	Status	1805 (12.83)	
	Photo	3280 (23.31)	
	Video	481 (3.42)	
	Link	7666 (54.48)	
	Event	839 (5.96)	
**Health message, n (%)**			
	Pre-exposure prophylaxis	119 (0.80)	
	Stigma	305 (2.20)	
	Mental health	180 (1.30)	
	Treatment	215 (1.50)	
	Testing	403 (2.90)	
	Research	380 (2.70)	
	Condoms	241 (1.70)	
	Dating	253 (1.80)	
**Time of week, n (%)**			
	Weekday (Monday-Friday)	12,368 (87.90)	
	Weekend (Saturday and Sunday)	1703 (12.10)	
**Time of day, n (%)**			
	Before work (1:00 am-7:59 am)	241 (1.71)	
	During work (8:00 am-4:59 pm)	10,734 (76.28)	
	After work (5:00 pm-12:59 am)	3096 (22.00)	
**Post feature**			
	Sentiment, median (IQR^a^)	0.09 (0.00-0.27)	
	Questions, n (%)	2824 (20.10)	
	Direct invitations to participate, n (%)	2326 (16.50)	

**Table 3:** Factors associated with user engagement.

Predictors of “engagement”		Regression coefficient B (SE)	Incidence rate ratio	*P* value
**Post type**				
	Status	Reference		
	Photo	1.10 (0.04)	3.00	<.001
	Video	0.84 (0.07)	2.32	<.001
	Link	0.51 (0.04)	1.66	<.001
	Event	–0.36 (0.06)	0.70	<.0001
**Health message (yes vs no)**				
	Pre-exposure prophylaxis	1.29 (0.12)	3.64	<.001
	Stigma	0.47 (0.08)	1.60	<.001
	Mental health	0.42 (0.11)	1.52	<.001
	Treatment	0.16 (0.10)	1.17	.10
	Testing	0.14 (0.07)	1.15	.06
	Research	0.01 (0.08)	1.01	.90
	Condoms	–0.06 (0.10)	0.94	.55
	Dating	–0.33 (0.09)	0.72	<.001
**Time of week**				
	Weekday (Monday-Friday)	Reference		
	Weekend (Saturday and Sunday)	0.07 (0.04)	1.07	.049
**Time of day**				
	Before work (1:00 am-7:59 am)	Reference		
	During work (8:00 am-4:59 pm)	–0.27 (0.09)	0.76	<.001
	After work (5:00 pm-11:59 pm)	–0.23 (0.09)	0.79	.01
**Post feature (yes vs no)**				
	Sentiment	0.52 (0.05)	1.68	<.001
	Questions	–0.10 (0.03)	0.90	<.001
	Direct invitations to participate	–0.06 (0.03)	0.94	.07

**Figure 2:**
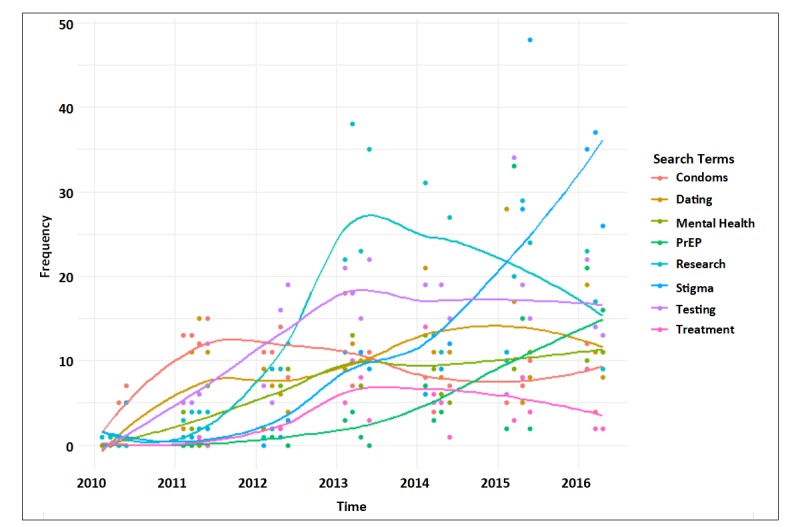
Loess smoothed mention of health messages overtime (2010-2016), stratified by keyword. PrEP: pre-exposure prophylaxis.

Post characteristics associated with user engagement are shown in Table 3. Higher user engagement was positively associated with positive sentiment (IRR 1.68), sharing photos (IRR 3.00), videos (IRR 2.32), and links (IRR 1.66), and posting about PrEP (IRR 3.64), stigma (IRR 1.60), and mental health (IRR 1.52). Figure 2 shows the frequency of health messaging over time for the key terms assessed in this analysis. Engagement was negatively associated with asking a question (IRR 0.90), posting about dating (IRR 0.72), sharing posts during (IRR 0.76) or after work (IRR 0.79) compared to before work and with sharing events (IRR 0.70).

## Discussion

This study collected post data from 10 Facebook pages promoting health or health-related events to GBM in Vancouver, BC. Together, these 10 pages had approximately 24,000 followers, shared approximately 14,000 posts, and amassed more than 25,000 engagements (ie, likes, comments, shares) during the 7 years’ of data analyzed. Although our data do not speak empirically to the true network structure of Facebook’s gay communities in Vancouver, we can make several important inferences regarding the network structure that underlies this analysis. First, based on the correlation between the number of groups and the number of engagements, our results point to the existence of a core group of users who may promote the diffusion of health content. Indeed, only a minority (38.2%) of users engaged more than once over the 7-year period we studied. These observations suggest that the true Facebook network structure of Vancouver’s gay community is indeed scale-free, as shown by Silenzio et al [25]. Second, because most users only engaged once over the extended timeframe of this analysis, our findings also suggest that shared content is broadly diffusing into distal regions of the network among individuals who may not be directly linked to the Facebook pages included in this analysis [28]. Third, as we observed modularity in user-post engagement, our findings also suggest that the 10 Facebook pages included in this analysis are serving multiple, distinct, although linked, clusters. Indeed, although some Facebook pages overlap in their outreach, our findings (see Figure 1) suggest that the combined effort of these organizations reaches into distinct user communities. This suggests that both strong and weak ties make the Facebook platform an ideal location for the diffusion of health content [27].

Our analysis also identified several factors that may enhance the diffusion of health content by increasing user engagement. These findings may be of help to CBOs because, unlike social network factors, they are amenable to intervention and change. For instance, we found that posts shared in the morning diffused better than those shared during working hours or after work. These results are consistent with previous studies that showed that posts can be strategically timed to take advantage of when users are active. Similarly, the richness of posts was also shown to be an important covariate of user engagement with higher engagement associated with photos, videos, and links, and lower engagement associated with sharing events. This is consistent with previous research [21] and with media richness theory [36], which suggests that “richer” media (ie, those with greater ability to efficiently convey messages, social cues, personalization, and feedback) better engages target audiences.

However, contradicting this theory, we also found that specific strategies to engage users, such as asking questions, were associated with lower user engagement. This supports other research that shows that inviting engagement, ironically, may be a less effective way to promote engagement [23]. Other research has shown more generally that traditional marketing elements discourage user engagement on Facebook [22]. This may reflect a distrust for traditional marketing and a desire for more authentic communication [37]. Indeed, Fromm et al [38] recommend that marketers approach younger audiences not as target populations, but as partners in the advertising process. Consistent with this approach, social media strategies should identify ways to authentically promote health with, not to, GBM [21]. Posts with positive affect did elicit higher engagement—perhaps reflecting the well-documented heuristic bias toward positive messaging [23,39].

Closely related to the form of posts, the content of posts was also seen to have a significant effect on user engagement. Posts about PrEP, stigma, and mental health exhibited greater engagement, whereas posts about dating had lower engagement. Although it is difficult to assess why some subjects engaged users better in this research, these finding may reflect the health priorities, or perhaps current controversies, in gay communities. Therefore, higher user engagement is expected when pages are posting content that might be trending and amenable to gay communities—highlighting the importance of community-conscious agendas for health promotion. Indeed, during the time of this study, community-driven campaigns around PrEP [40] and stigma [41] may have served as driving forces behind user engagement with posts regarding PrEP, stigma, and mental health. Conversely, posts relating content regarding HIV-related behaviors (eg, testing and condoms) seemed to attract fewer engagements, potentially highlighting the difficulty of using social media to promote well-established prevention strategies. This may be particularly true for those with which audiences have become fatigued, such as has long been reported among GBM in San Francisco [42]. Based on our results, future analyses should investigate whether integrating better diffusing content, such as PrEP and stigma, into posts promoting more traditional prevention strategies has the potential to improve the diffusion of this content.

Regular assessment of how users are engaging with posts relevant to specific key themes may provide public health and community leaders with insight into the diffusion of social discourse surrounding important topics of concern. To this point, we note significant temporal variation in the frequency at which key themes were included in CBO posts. As mentioned before, PrEP and stigma increased throughout the observation period likely due to specific prevention campaigns in Metro Vancouver. Similarly, the frequency at which research and testing were discussed increased dramatically during the first half of the observation period, with research-related posts peaking in early 2013 and declining thereafter, and testing-related posts leveling off at the same time. Because this study was primarily focused on engagement and not the CBO’s rationale for content selection, future studies might improve our understanding of what factors contribute to the ebb and flow of specific key themes.

Further, future research should examine individual-level data, particularly that of core users, whom our findings suggest may play an important role in the diffusion of post content. Such examinations might be conducted by each CBO because they may have greater access and interest in these specific analyses. More generally, our findings also highlight the importance of the user experience in shaping the diffusion of health content. Therefore, ongoing cooperation with users is needed to identify the features that should be leveraged in health promotion—especially because users, not social media specialists, are the ultimate arbiters of whether content is shared with their networks. Consistent with this, CBOs may benefit from examining the network dynamics of their followers and leverage the approaches used in this study to identify specific users who might be willing to partner with CBOs to promote their content.

These findings should be interpreted with consideration of the limitations of this study. First, because CBOs were not selected using a randomized approach, it is difficult to say whether our findings are generalizable to all Facebook-based health promotion efforts. However, we included most of the major pages associated with organizations in Metro Vancouver. Therefore, our results best represent the health priorities of Vancouver’s gay community, although they may not be the same as those in other communities. Second, because we used relatively simple informatics-based analytic approaches to identify and code posts, our analysis is subject to measurement error. In particular, the selection of key terms may limit the accurate classification of posts relevant to the post features and health messages we explored. However, based on the consistency of our findings with studies conducted regarding other health areas, it seems that our approach produced similar results to studies that included manual coding techniques [22,23]. Nevertheless, validation of the results of this study is needed, both in other geographic settings and with other sexual or gender minority communities. Third, because the engagement factors for Facebook reactions, comments, and shares may differ [21,23], further analysis is needed on how to elicit the type of participation that will best promote health awareness. This is especially important given that the predictors of likes, comments, and shares may not be the same. Indeed, because we summed across these three types of user engagement, we may be obscuring important differences or patterns. For example, posts that elicit comments may elicit fewer shares, thus misestimating user engagement with shared posts. Furthermore, Facebook’s EdgeRank algorithm, which determines whether content is diffused and shown on people’s Facebook pages, is constantly updated and the relative weighting of various types of interaction may change, making it important to understand the unique determinants of various types of engagement (ie, reactions, comments, shares). Future analyses should expand our findings by evaluating the factors associated with specific engagement indicators. Lastly, other important factors, which we have not considered, may also shape user engagement. These include individual-level factors, which require a different analytic and sampling approach to understand how specific user characteristics may shape user engagement. Although engagement at the individual level is difficult to study, integrating Facebook plug-ins into study questionnaires might allow researchers to match social media participation to survey responses. Other important considerations may also include specific factors that might persuade different individuals to engage with post content, underscoring the need for further examination of gay and bisexual men’s social media engagement. Likewise, exploration of additional themes that were not examined in this analysis is needed. Indeed, only a minority of posts were relevant to the themes we selected and examined. Undoubtedly, CBOs have interest in sharing and promoting content that may not necessarily be directly related to health outcomes studied by public health researchers. Despite these limitations, this study supports the use of Facebook for health promotion among sexual minorities and highlights multiple factors that can be leveraged to optimize user engagement, thus enhancing the diffusion of health information and the reach of CBOs.

## Abbreviations

API: application programming interface
CBO: community-based organization
GBM: gay, bisexual, and other men who have sex with men
IRR: incidence rate ratio
PrEP: pre-exposure prophylaxis
